# Inherited thrombophilia transpires with severe coronary arterial thrombosis in wide range of age backgrounds: A report of 3 cases

**DOI:** 10.1016/j.amsu.2022.103730

**Published:** 2022-05-10

**Authors:** Mohammad Satya Bhisma, Iswanto Pratanu, Ryan Enast Intan, Firas Farisi Alkaff

**Affiliations:** a Department of Cardiology and Vascular Medicine, Faculty of Medicine Universitas Airlangga – Dr. Soetomo General Academic Hospital, Jl. Mayjend Prof. Dr. Moestopo No 4–6, Surabaya, East Java, 60286, Indonesia; b Division of Pharmacology and Therapy, Department of Anatomy, Histology, and Pharmacology, Faculty of Medicine Universitas Airlangga, Jl. Mayjend Prof. Dr. Moestopo No 47, Surabaya, East Java, 60132, Indonesia; c Division of Nephrology, Department of Internal Medicine, University Medical Center Groningen, Hanzeplein 1, Groningen, 9713, GZ, the Netherlands

**Keywords:** Case report, Myocardial infarction, Thrombophilia, Protein C, Protein S

## Abstract

**Introduction:**

and importance: Protein C and S deficiency are some of the coagulation cascade disorders which may also contributes not only to venous thromboembolism (VTE), but also rarely to arterial thrombosis. Here we present a report of 3 severe coronary artery disease (CAD) cases ranging from very young to elderly patients with concomitant inherited thrombophilia.

**Case presentation:**

The first case was a chronic coronary syndrome from a very young male patient with history of VTE without any other risk factor of CAD. The second case was about premature CAD with triple chronic total occlusion (RCA, LCX, LAD) in patient under 45 years old, with single risk factor. The third case was about accelerated atherosclerosis progression from previously non significant stenosis in RCA into total occlusion in RCA and inferior STEMI in old patient on supposely adequate double anti platelet agent.

**Clinical discussion:**

All patients had protein C and/or S deficiency and first degree family history of VTE, therefore inherited thrombophilia was diagnosed. We gave them oral anticoagulant in addition to their standard treatment for secondary prevention with good outcome and without further adverse event.

**Conclusion:**

It is important to raise awareness to perform screening inherited thrombophilia as an important risk factor for CAD in special subgroup such as young age patient with rapid course progression and family history of VTE. The use of oral anticoagulants as either prophylactic or therapeutic purpose in patients with inherited thrombophilia are safe and effective. However, further research is still needed.

## Highlights

• Inherited thrombophilia is a rare disease that can causes coronary arterial thrombosis.
• Inherited thrombophilia screening should be done in special subgroup of patients.
• There is no treatment guideline for CAD patients with inherited thrombophilia.

## 1 Introduction

Inherited thrombophilia, a collection of hereditary diseases that predispose to thrombosis and are characterized by heritable deficits of the endogenous anticoagulants proteins and antithrombin, is linked to an increased risk of thromboembolism. It can potentially be the cause of coronary artery disease (CAD) and myocardial infarction (MI), especially in young adults [1]. One of the most common variants of the thrombopohilia is because of factor V Leiden gene mutation, which results in resistance to protein C inactivation. Other causes of inherited thrombophilia include protein C, protein S, and antithrombin deficiency [1].

Previous study suggested that thrombophilia is an independent risk factor for CAD in young patients [2]. However, the specific pathophysiology remains unclear. Further, there is currently no treatment guideline for people with thrombophilia and atherosclerosis who have acute coronary syndrome [3]. Here we present 3 cases of inherited thrombophilia in different age groups, in which all of them had severe coronary arterial thrombosis, and treated with different treatment modality. This case report has been reported in line with the SCARE Criteria [4].

## 2 Presentation of case

### 2.1 Case I

A 26-year-old-man was referred to our hospital for a follow up evaluation for anteroseptal acute myocardial infarction (AMI) that occurred 3 months ago. The patient still felt occasional chest pain which exaserbated by physical activity. The vital sign when the patient came was stable. When the anteroseptal AMI occurred, the patient was treated with conservative medications due to the limited resources in the previous hospital. Laboratory results at that time were as follows: Fibrinogen level 433, total cholesterol 176 mg/dl, LDL 101 mg/dl, and triglycerides 225 mg/dl, responsive Aspirin and Antiplatelet Response Test (431 amd 104, respectively), and Platelet aggregation test (PAT) result was 23/22/46. The patient was consuming various oral medications after the recent AMI, i.e., Aspirin 100 mg once daily, Clopidogrel 75 mg once daily, Trimetazidine 35 mg twice daily, Perindopril 5 mg once daily, Pravastatin 20 mg once daily, Bisoprolol 5 mg once daily, isosorbit dinitrate 5 mg twice daily, and warfarin 2 mg once daily.

Three years ago the patient had Deep Vein Thrombosis (DVT) in right inferior extremity with INR of 1.8 (therapeutic target 2.0–3.0) and treated with vitamin K antagonist (VKA). History of coronary artery diseases (CAD) in the family was denied. However, the father has a medical history of venous thromboembolism (VTE).

ECG evaluation revealed a sinus rhythm of 85 beats per minute (bpm) on the frontal RAD axis, horizontal clock wise rotation (CWR), and Anteroseptal old myocardial infarct (OMI). Further examination by Exercise Stress Test (EST) with BRUCE protocol on treadmill showed normal findings with 10 metabolic equivalents (METS). Echocardiography evaluation showed left ventricular (LV) dilatation (left ventricular internal end-diastolic diameter 5.8cm), myocardial thinning, decrease LV systolic function (LV ejection fraction 44.9%), and hipokinetic anteroseptal. The patient was suspected for chest pain secondary to CAD, with differential diagnosis of VTE. Nevertheless, the patient's condition at that time did not meet the criteria for VTE based on Geneva pre test probability score.

**Fig. 1:**
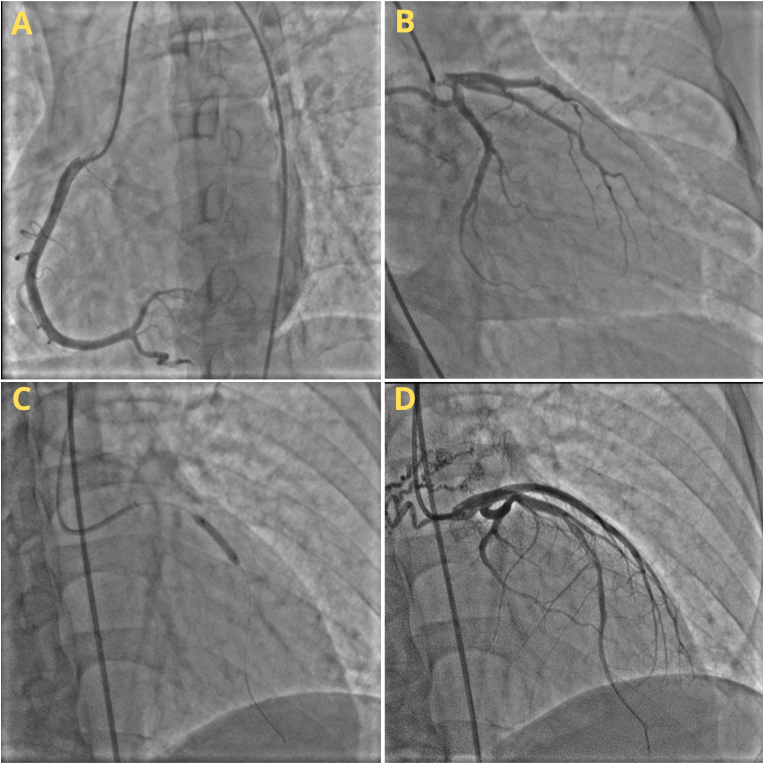
Coronary Angiography and Percutaneous Interventions of Case I: (a) RCA, (b) LMCA, LAD, and LCX, (c) Side-by-side placement of a stent in the mid-LAD, (d) the catheterization's final result.

Patients then underwent coronary angiography and percutaneous intervention, which was operated by an interventional cardiologist. A 80–90% stenosis was found in the mid left anterior descending artery (LAD) with clotting intra-coroner, and a small caliber in the distal LAD. No stenosis was present in other arteries. After that, 3.5/18 and 3.0/18 Absrob™ dissolving stents were installed side-by-side along the Mid LAD (Fig. 1). The procedure went well, and the patient was in good condition without any significant complaint after the intervention.

After the intervention procedure, the patient was screened for coagulation disorder by testing the protein C and protein S because it was rather unusual to have CAD and VTE without significant risk factor in young age. Prior to the testing, the VKA medication was stopped for 3 weeks to minimize false result. The test result showed protein C deficiency (54 IU/dL, reference value 70–140 IU/dL) and normal protein S (105 IU/dL, reference value 70–140 IU/dL with history of VTE). The patient was then diagnosed with a inherited thrombophilia with protein C deficiency. The patients was given direct oral anticoagulant (DOAC) (rivaroxaban 10 mg) once daily since then. The VKA medication was discontinued, but other oral medications were continued to be given. Until two years afterward, the patient had no symptoms, side effect, or complications.

### 2.2 Case II

A 44-year-old-man came to a cardiologist private clinic with a chief complaint of chest pain, which was felt since 1 year ago, and gotten more frequent since 1 month prior. The chest pain spread to the neck and left arm and aggravated with activity. The patient also had dyslipidemia (total Cholesterol 225 mg/dl, triglyceride 194 mg/dl, high density lipoprotein 33 mg/dl, and low-density lipoprotein 168 mg/dl). History of hypertension, diabetes, and smoking were denied. Family history of CAD was denied; however, the first-degree family had DVT previously. EST 6 months ago revealed significant horizontal ST depression in lead V3–V6 at peak and post-exercise.

The patient's vital signs were stable (BP 130/80, HR 70x/min, RR 20x/min). The physical examination showed positive S4 heart sound without any murmur or crackles or other sign of congestion. ECG examination showed normal sinus rhythm with normal axis. Chest X-ray showed normal findings with cardio-thoracic ratio of 40%. Echocardiography evaluation showed normal heart function and structure with EF teich of 67%. Patient was diagnosed with CAD, with routine treatment of clopidogrel 75 mg once daily, Atorvastatin 40mg once daily, and Bisoprolol 2.5mg once daily.

**Fig. 2:**
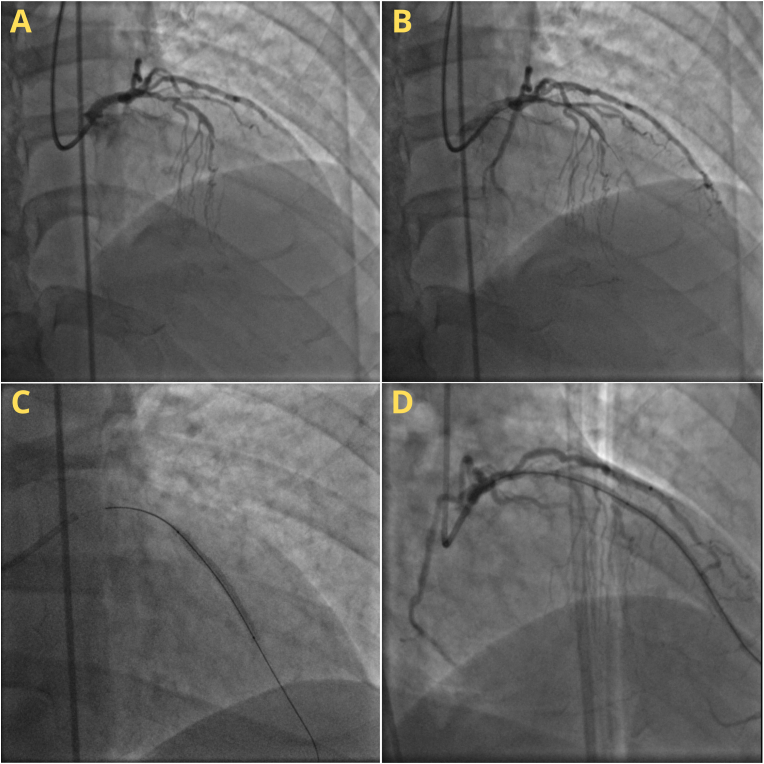
Coronary Angiography and Percutaneous Intervention of Case II: (a) LMCA, LAD, and LCX, (b) Wire through the CTO LAD lesion, (c) Stent in Proximal – Mid LAD, (d) End result of catheterization.

The patient was then referred to our center to undergo coronary angiography and percutaneous intervention. The procedure was done by an interventional cardiologist. Chronic total obstruction (CTO) in mid LAD and mid right coronary artery (RCA), significant stenosis (70%) in proximal LAD, branch D1 with diffuse stenosis (50–70%) with collateral level II towards distal left circumflex artery (LCX), bridging collateral level II from septal branch to distal LAD, and CTO in distal LCX with collateral level II from LCX distal and first obtuse marginal artery (OM1) branch to Right posterior descending artery (RPDA) and right posterolateral artery (RPL) branches were found. Based on the findings, the patient was diagnosed with CAD triple CTO in RCA, LCX, and LAD. STENT XLIMUS 2.75/40 and PROMUS 3.5/20 were then placed in proximal to mid LAD (Fig. 2). After the procedure, the patient only felt a little discomfort that gradually resolved.

However, there was a suspicion of an underlying coagulopathy abnormality because of the CAD severity with the presence of 3 CTOs despite the lack of significant cardiovascular risk factors. Thus, protein C and protein S were checked, and it was found that there was the protein C (48 IU/dL, reference value 70–140 IU/dL) and protein S (54 IU/dL, reference value 65–130 IU/dL without history of VTE) were deficient. Inherited thrombophilia with protein C and Protein S deficiency was then concluded as the diagnosis of this patient.

Other than that, there was also a suspicion of thrombocyte aggregation abnormality as the differential cause. Therefore, PAT was done, and the test result was 15/13/57. The patient was then treated with several oral medications, i.e., Aspirin 80 mg once daily, clopidogrel 75 mg once daily, atorvastatin 40 mg once daily, bisoprolol 2.5 mg once daily, and warfarin 2mg once daily. INR examination 1 month afterward showed therapeutic range result (2.2, therapeutic target 2.0–3.0). Warfarin dose was then continued with routine monitoring of INR every 3–6 months. Until after 1 year, the patient showed no symptoms or complications.

### 2.3 Case III

A 62-year-old man came with a chief complaint of typical chest pain that radiates to the left arm and triggered by heavy activity or exercise since 3 month prior. The patient had a history of hypertension and dyslipidemia. The patient also smoke cigarette 80 pack years. The first degree family had past medical history of CAD and VTE.

**Fig. 3:**
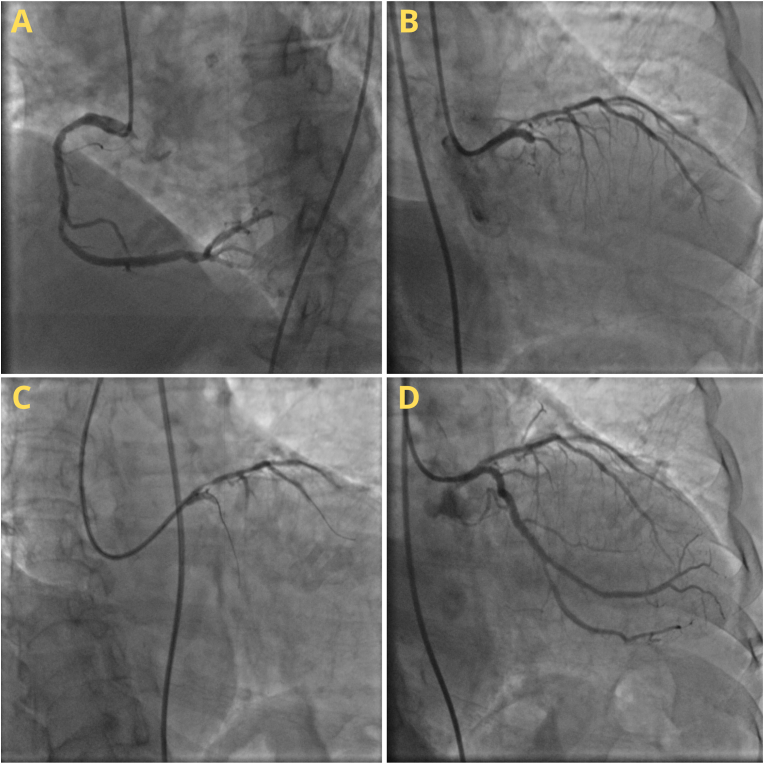
Coronary Angiography and Percutaneous Intervention of Case III: (a) RCA, (b) LMCA, LAD, and LCX, (c) Wiring in LAD and LCX, (d) Post stent placement on LAD and LCX.

The patient's physical examination was unremarkable with stable vital sign (BP 140/80, HR 70 bpm, RR 16x/min). ECG evaluation showed QS pattern in V1–V2 and T-inversion in V2–V4. Echocardiography evaluation showed normokinetic LV and RV but slightly decreased LV systolic function (LVEF 51.3%). The patient was suspected of CAD, with differential diagnosis of VTE. The patient then underwent elective coronary angiography that was done by an interventional cardiologist. The angiography findings were as follows: Left main coronary artery (LMCA) minor disease, total occlusion in proximal LAD with collateral from the conus branch, CTO in proximal LCX with collateral grade-3 from RPL to LCX, and a large caliber RCA with non-significant diffuse stenosis (30–50%) along proximal-mid RCA. The Cordis 2.5/33 stents was then placed on proximal LAD and ORSIRO 3.0/30 stents was placed on LCX (Fig. 3). The PAT result afterward was 69/0/64. The patient then received Aspirin 80 mg once daily, clopidogrel 75 mg once daily, bisoprolol 2.5 mg once daily, and atorvastatin 40 mg once daily.

A year later, the patient experienced a severe typical angina attack, with normal physical findings and stable hemodynamic. ECG examination showed ST elevation in inferior lead (II, III, AVF) and ST depression in lead I, AVL, V5–V6. The patient was then diagnosed with inferior STEMI and treated with thrombolytic (streptokinase 250,000 IU) in 30 minutes. However, the patient showed an allergic reaction, so the thrombolytic treatment was stopped. Post thrombolytic ECG showed QS pattern in lead III and AVF, and ST Depression in lead I, AVL, V5–V6. The patient was then treated conservatively.

Six month later, the patient underwent elective coronary angiography. A patent old stent in proximal LAD and proximal LCX with collateral grade-3 from RPL to LCX, and CTO on RCA distal with bridging collateral grade II-III from the RV branch to the RCA distal were found. Then, a lesion penetration attempt was conducted with GW Whisper wire and GW Runthrough; however, the wire was unable to penetrate the lesion, and the attempt was stopped. The patient was in a good and stable condition after the procedure, and just felt a little discomfort of the angina.

To investigate the abnormality over the rapid occlusion progressivity in RCA, protein C and Protein S test were done to screen the possible coagulopathy abnormality. The patient showed to have protein C deficiency(65 IU/dL, reference value 70–140 IU/dL) and normal Protein S (67 IU/dL, reference value 65–130 IU/dL without history of VTE). The patient was then diagnosed with mixed inherited and acquired thrombophilia with protein C and Protein S deficiency. The patient was then treated with warfarin 2 mg once daily and titrated until reached the INR therapeutic range (2.0–3.0) in addition to the previous medications that were still being given. Until after 6 months, the patient had well tolerated symptom (CCS class II) with no side effect nor complications. Afterward, the patient was then referred back to the secondary referral hospital for routine control.

## 3 Discussion

In this case report of 3 patients, we presented unusual course of CAD spectrum from young age to older age which might be related to inherited thrombophilia. All three cases shared some similarities, i.e., all had a history of VTE from their respective first degree relative and had protein C and/or protein S deficiency. Summary of the patients are presented in Table 1.

**Table 1:** Summary of the reported cases.

Cases No.	Sex and age	Arterial thrombosis event	Coronary angiography	History of VTE	First degree Family history of VTE	Protein C or protein S deficiency	CV risk factors	Thrombophilia possibility	Anti-coagulant prophylaxis
I	Male, 26 years old	MI	LAD stenosis 80%	yes	yes	Protein C	none	Inherited thrombophilia	DOAC
II	Male, 45 years old	–	Triple CTO	No	Yes	Protein C and protein S	Dyslipidemia only	Inherited thrombophilia	VKA
III	Male, 62 years old	MI	CTO in LAD and LCX, non significant stenosis in RCA that progressed to CTO in 1 year	No	Yes	Protein C and protein S	Hypertension, smoking, dyslipidemia, family history CVD	Mixed thrombophilia	VKA

CVD, cardiovascular disease; CTO, chronic total obstruction; DOAC, direct oral anticoagulant; LAD, left anterior descending coronary artery; LCX, left circumflex; MI, myocardial infarction; RCA, right coronary artery; VKA, vitamin K antagonist; VTE, venous thromboembolism.

The prevalence of inherited thrombophilia in general population is very rare (0.25–0.5%). However, there is a significant increase in the distribution of up to 10 times in the VTE population. Inherited thrombophilia is an uncommon risk factor that is often overlooked or ignored, apart from its very subtle manifestations, which are often only discovered when the first vascular event occurred. Not only manifest as VTE, inherited thrombophilia can also manifest in arterial thrombosis including CAD or MI, although it is very rare [2]. Of all inherited thrombophilia and their variants, protein C and protein S deficiency are a very strong predictor of the atherothrombotic disease incidence in young people that can increased the risk of MI for 8.76 times for protein C deficiency and 3.2 times for protein S deficiency [5].

There are some evidences that show strong association between protein C and protein S deficiency with VTE and arterial thrombosis. Baglin et al. (2010) found a 10–15x increased risk of VTE in patients with protein C and S deficiency, and 75% of them also have family history of VTE especially in their 1st degree relatives, coining the term inherited thrombophilia [6]. Meanwhile for arterial thrombosis, the incidence of inherited thrombophilia is less common in general population [7]; however, it was higher in STEMI in young adults. A retrospective observational study of thrombophilia screening in STEMI patients under 55 years old showed an abnormality in 43% of cases, and they presented with less cardiovascular risk factors than patient without thrombophilia investigation (p < 0.001) [8]. Our cases also added more evidence that inherited thrombophilia can be found in arterial disease, especially in young patients (case 1 and case 2), and with unusual course of event (triple CTO in less than 45 year old patient (case 2), and also rapid progressive to CTO in just 1 year (case 3).

Protein C and protein S are vitamin K-dependent anticoagulant proteins synthesized in the liver, and their deficiency are associated with a minority of cases of inherited thrombophilia. Protein C circulates as a zymogen and performs its anticoagulant function after being activated to become activated protein C (aPC), which functions to inactivate coagulation factors Va and VIIIa, which play a role in thrombin formation and activation of factor X. The inhibitory effect of aPC is enhanced by protein S, resulting in protein deficiency. Protein C and protein S can cause thrombophilia, which is an increased tendency to thrombose. Patients with hereditary protein C and S deficiency lack the natural anticoagulant function of activated protein C and are at risk for clinical phenotypes associated with an increased risk of thrombosis including VTE, stroke, and CAD [7,9,10].

The diagnosis of inherited thrombophilia is established from a series of laboratory tests. However, the facility needed for test factor V Leiden is still very rare in Indonesia because it requires genetic DNA testing, or the aPC resistance Assay [11]. The option which is more widely available in Indonesia for thrombophilia testing is through the examination of protein levels C and S. Because inherited thrombophilia is very rare and the results do not necessarily provide significant changes in treatment and therapy, only patient with high-risk thrombosis is eligible for thrombophilia screening, e.g., patients who experienced VTE or arterial thrombosis in young age and have family history of VTE under the age of 45 years, especially 1st degree relatives [[11], [12], [13]].

Several clinical conditions and the use of anticoagulants can affect the results of the examination. Protein C and protein S deficiency cannot be determined in patients taking DOAC and warfarin. If testing is still required, treatment is changed to full-dose heparin or LMW heparin, and warfarin is discontinued for at least 3 weeks prior to measurement (as in our first patient). The amount of protein S and antithrombin (AT) can decrease in acute thrombotic conditions, so it is better not to do the examination in acute conditions [14,15]. Hence, in these case reports we chose to test the protein C and S screening 3–6 months after acute events when the patients are in stable condition.

Treatment that can be given to patients with protein C deficiency who experience thromboembolism is anticoagulation. The choice between DOAC or warfarin is based on a number of factors including the severity of the thrombosis, patient preference, adherence to therapy, and potential drug and dietary interactions. A 5-year prospective cohort study that evaluated the efficacy and safety of DOAC compared with heparin/VKA in acute VTE with inherited thrombophilia showed that the use of DOAC showed an equivalent efficacy profile compared to heparin/VKA in terms of the treatment of thrombosis, but DOAC was shown to be safer for use in patients with thrombophilia and was associated with a significant reduction in VTE recurrence in the follow-up period of 2 years after discontinuation of therapy [16].

In our cases, the first patient was already on routine VKA treatment when he was having MI, so we opted to change the anticoagulant with DOAC for further thromboprophylaxis. Meanwhile for the second and third case, VKA was given with monitoring of INR and side effect such as warfarin-induced necrosis. However, the duration of anticoagulant for thromboprophylaxis after arterial thrombosis event in patient with inherited thrombophilia is not known, because there is still no established guideline about it until now. The only current available recommendation is to give anticoagulants for thromboprophylaxis in inherited thrombophilia patients with unprovoked VTE and with first degree family history of VTE for at least 3–6 months to extended indefinite duration of time based on individualized cases [17], therefore we adopt this recommendation in our patients with arterial thrombosis to prevent further MI secondary to inherited thrombophilia.

In this report, there are 2 important limitations. First, evaluation of factor V Leiden as the most common cause of inherited thrombophilia cannot be done. Second, first-degree relatives who had a history of VTE at a young age were not screened for protein C and S deficiencies because of financial limitations. However, our report was the first that show a probable relation between protein C and S deficiency with arterial thrombosis in young adult and unusual rapid progressivity, along with the comprehensive management.

## 4 Conclusion

Inherited thrombophilia is a rare condition but might also serve as an important risk factor for CAD in special subgroup such as young age patient with rapid course progression and family history of VTE. Therefore, it is important to perform screening for inherited thrombophilia in selected cases such in our report. The use of oral anticoagulants as either prophylactic or therapeutic purpose in patients with inherited thrombophilia are considered safe and effective, while further researchs are still needed.

## Learning points

• Inherited thrombophilia is a rare phenomenon in general population and often undetected; however, it can lead to many manifestations including coronary arterial thrombosis.
• In patients with rapid progressive of coronary artery disease, chronic total occlusion in younger age, or with history of previous venous thromboembolic or history of first-degree family history of venous thromboembolic, inherited thrombophilia should be considered as one of the underlying diseases. Thus, protein C and protein S level should be checked in these patients.
• Treatment of coronary artery disease with inherited thrombophilia includes usual standard therapy and may need the addition of anticoagulant, with warfarin or direct oral anticoagulant, with individualized duration of treatment based on the patient's condition and side effect monitoring.

## Ethical approval

N/a.

## Sources of funding

None.

## Author contribution

MSB contributed to the data collection and drafted the initial manuscript. IP contributed to the conceptualization and design of the study and drafted the initial manuscript. REI and FFA contributed to the data analysis and interpretation and revised the manuscript for important intellectual content.

## Research registration

N/a.

## Guarantor

MSB and IP are the guarantor for this case report.

## Consent of patients

Written informed consent was obtained from the patient for the publication of the case report and the accompanying images. A copy of the written consent is available for review by the Editor-in-Chief of this journal on request.

## Provenance and peer review

Not commissioned, externally peer-reviewed.

## Declaration of competing interest

The authors declare that they have no conflict of interest.

## Declaration of competing interest

None declared.
